# Increased body mass index (BMI) associated with late epilepsy onset in patients with temporal encephaloceles: a systematic review and analysis of individual patient data

**DOI:** 10.1186/s42466-024-00329-0

**Published:** 2024-07-11

**Authors:** Panagiota-Eleni Tsalouchidou, Hans-Helge Müller, Ioannis Mintziras, Sascha Strehlau, Wiebke Hahn, Marcus Belke, Felix Zahnert, Gunter Kräling, Katja Menzler, Susanne Knake

**Affiliations:** 1 Epilepsy Center Hessen, Department of Neurology, Philipps University Marburg, 35043 Baldingerstrasse, Marburg, Germany; 2 Institute of Medical Bioinformatics and Biostatistics, Philipps University Marburg, Marburg, Germany; 3 Department of Visceral-, Thoracic- and Vascular Surgery, Philipps University Marburg, Marburg, Germany; 4 Second Department of Neurology, Attikon University Hospital, National and Kapodistrian University of Athens, Athens, Greece

**Keywords:** Temporal lobe epilepsy, Temporal encephaloceles, Body Mass Index (BMI), Structural epilepsy, Seizures

## Abstract

**Objective:**

This study investigates the association of Body Mass Index (BMI) and age of epilepsy onset, in patients with epilepsy associated with temporal encephaloceles (TEs).

**Methods:**

A comprehensive PubMed literature review was conducted using the keywords “temporal encephaloceles” and “epilepsy” for identifying articles for the analysis. Inclusion criteria encompassed all evidence levels reporting patients with TE-related epilepsy and documented BMI. Logistic regression analyses were performed to examine the effect of BMI on predicting epilepsy onset after the 25th year of age. Spearman’s correlation assessed the relationship between BMI with epilepsy onset. Finally, the association between BMI and postsurgical outcomes, distinguishing between more favourable outcomes (Engel Class I and II) and less favourable outcomes (Engell Class III and IV) was explored.

**Results:**

Of the initially identified 88 articles, nine were included in the analysis, involving 127 patients with TE-related epilepsy and reported BMI. The mean age of epilepsy onset was 24.9 years (SD = 14.8 years), with a mean BMI of 28.0 kg/m^2^ (SD = 7.4 kg/m^2^). A significant positive correlation was observed between BMI and age of epilepsy onset (rho = 0.448, *p* < 0.001). Female patients had higher BMI compared to male patients (30.1 kg/m^2^, SD = 8.7 kg/m^2^ and 26.5 kg/m^2^, SD = 5.3 kg/m^2^ respectively, *p* = 0.008). However, the epilepsy onset did not differ significantly between male and female patients (*p* = 0.26). The bivariate logistic regression showed that patients with increased BMI were more likely to have an epilepsy onset after the 25th year of age, adjusted for the confounder sex (OR = 1.133, 95%-CI [1.060, 1.211], *p* < 0.001). Finally, a potential trend indicated a higher average BMI among patients with more favourable postsurgical outcomes than less favourable postsurgical outcomes (27.3 kg/m^2^, SD = 7.7 kg/m^2^ and 24.8 kg/m^2^, SD = 2.2 kg/m^2^ respectively, *p* = 0.076).

## Significance

The occurrence of TEs has already been associated with increased BMI and idiopathic intracranial hypertension. The current analysis indicates a significant correlation between elevated BMI and delayed epilepsy onset in cases of TE-related epilepsy. This finding suggests the presence of unique underlying pathomechanisms among epilepsy patients with higher BMI and TEs, supporting the hypothesis of a potential gradual influence of BMI on TE formation. Consequently, this may lead to a milder, more gradual progression of epileptogenesis compared to individuals with a normal BMI.

## Introduction

Temporal encephaloceles (TEs) are protrusions of brain parenchyma through skull base or cranial vault, commonly associated with several otorhinolaryngological and neurological conditions [1]. Their severity varies, with some being small and asymptomatic [2], while others may cause significant neurological symptoms such as cerebrospinal fluid (CSF) leakage and temporal lobe epilepsy. TEs have either a congenital or an acquired origin [1]. Congenital encephaloceles occur due to the failure of the neural tube to close properly during fetal development, leading to herniations of brain tissue, CSF, and meninges. They may be present at birth, often associated with other congenital abnormalities [1]. In contrast, acquired or secondary encephaloceles develop postnatally due to conditions affecting the dura and skull [1].

Several case series have demonstrated that increased intracranial hypertension and BMI are commonly observed in the occurrence of TEs. This association has been documented in both patients with and without epilepsy [3–5]. The exact underlying pathomechanism is not well understood. Increased BMI is associated with alterations in CSF dynamics and an increased risk of developing idiopathic intracranial hypertension (IIH), leading progressively to a gradual formation of the TEs [6]. Moreover, predisposing factors such as susceptibility of the skull base and the presence of abnormally thin or fragile skull base bones might contribute to the development of TEs and CSF leakage [7].

Remarkably, despite an elevated BMI being associated with developing TEs, several published case series report that most of the included patients have a normal BMI [8, 9]. As already suggested by previous studies [10], an elevated BMI influences the formation and further development of TEs, implying that the pathogenesis and the onset of epilepsy might differ between patients with and without this factor. Consequently, the question that arises is whether TE-related epilepsy presents distinct features in patients with increased BMI. To investigate possible unique patterns of TE-related epilepsy syndrome in patients with elevated BMI and to examine the underlying pathomechanisms and potential associations, we conducted a broad literature review and subsequent analysis of patients reported in articles on TE-related epilepsy and documented BMI.

## Methods

### Literature search strategy, search terms and data extraction

We conducted a broad literature review based on PRISMA [11] guidelines to comprehensively explore a potential relationship between BMI and TE-related epilepsy. The search was restricted to articles published in English and available on PubMed, with the most recent search conducted in August 2023. To identify relevant studies, we employed the following keywords: “temporal encephaloceles” and “epilepsy”. The titles and abstracts of all identified citations were screened by two investigators (PT and IM) to determine their relevance. Then, the full texts of potentially relevant articles were retrieved and rescreened by the same authors (PT and IM) separately. In addition, the reference lists of those publications were reviewed (PT and IM) to identify further relevant articles suitable for inclusion.

### Inclusion and exclusion criteria

Articles reporting patients with TE-related epilepsy and their corresponding BMI were included in the analysis. For studies investigating the relationship between BMI and TE-related epilepsy syndrome, we specifically selected articles that provided individual BMI values for each included patient. Articles solely describing BMI in terms such as “obese” or presented BMI only as mean or median values were excluded from the analysis. Duplicate patient publications were included only once in the analysis. Articles published in languages other than English were excluded. All study designs were eligible for inclusion, regardless of the level of evidence, as the published articles on this topic predominantly consist of case series and case reports.

### Data extraction

The following data were extracted from each eligible study by two investigators (PT and IM): study design, year of publication, study location, total sample size, number of patients with reported BMI, patient demographics including sex and age of epilepsy onset, number of surgically treated patients with reported BMI and postsurgical outcome. Moreover, we collected data on patients who showed indirect signs of ΙΙΗ on MRI, such as an empty sella or had reported an increased CSF opening pressure.

### Statistical analysis

Statistical analyses were performed using SPSS version 23 (IBM, 2017). Categorical variables are presented as proportions, while continuous variables are presented as means followed by the standard deviation (SD). Separate univariate logistic regressions were conducted for BMI and sex, each considering the seizure onset before and after the 25th year of age as dependent variable. Subsequently, a bivariate logistic regression was performed, including both BMI and sex as predictors, with the same dependent variable representing seizure onset before and after the 25th year. To differentiate between an earlier and later age of epilepsy onset, we selected the 25th year of age based on the fact that brain maturation continues until about the age of 25 years [12]. There were no violations of the assumptions of the model (sample size, multicollinearity, outliers). Moreover, Spearman correlation analysis was conducted to assess the correlation between the seizure onset and BMI within the total population. Student’s t-test was used to compare the age of epilepsy onset between the male and female groups and between patients with and without signs of ΙΙΗ. For unequal variances, values of Welch´s t-test were reported as most appropriate. All tests were two-sided with a value of 0.05 set as the threshold for significance (in case of alternative with two directions 0.025 for each direction).

## Results

### Selected studies and patients’ characteristics

**Fig. 1:**
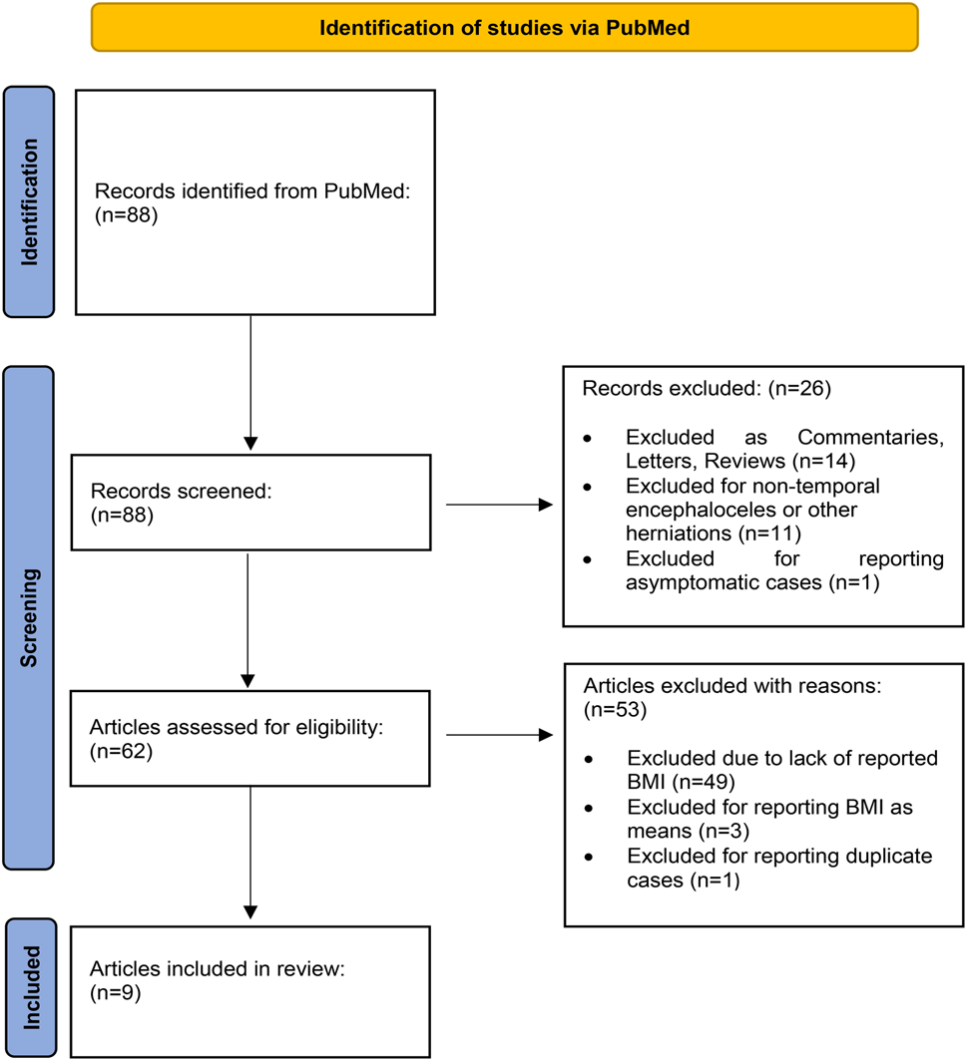
PRISMA flow chart describing the article selection process

A total of 88 articles were initially identified through the comprehensive literature review. Among these, 49 articles were excluded due to lack of reported BMI; three articles were excluded for reporting BMI as means; 14 articles were excluded for falling into categories such as Commentaries, Letters to the Editor and Reviews; one article was excluded for reporting asymptomatic individuals; 11 articles for reporting encephaloceles of non-temporal localization (such as frontal or occipital) or brain herniations other than TEs; one article was excluded due to duplicate records. Overall, nine articles were eligible for inclusion in the analysis [5, 8–10, 13–17]. The above mentioned exclusion criteria are presented in the PRISMA diagram in Fig. 1[11].

Of the nine included articles, a total of 127 patients with TE-related epilepsy and available BMI were included in the analyses. Of these patients, 48.0% (*n* = 61) were female, 44.9% (*n* = 57) were male, and in one article, 7.1% (*n* = 9) the information on sex was not reported [17]. The mean age of epilepsy onset was 24.9 years (SD = 14.8 years). The mean BMI of the included population was 28.0 kg/m^2^ (SD = 7.4 kg/m^2^, range = 16.2–53.3 kg/m^2^). The general characteristics of the included studies and patients are presented in Table 1.

Regarding the aetiology of TEs, explicit reporting on potential causes, such as trauma or infections, was not available in most of the included studies. Among these nine studies, three reported a positive history of head trauma [8, 10, 14]. In two of these studies, trauma was considered irrelevant to TE formation [10, 14], while in the third study [8], trauma was suggested as a potential cause in two patients. Additionally, none of the included patients had a documented history of infection as a probable TE cause. Due to the absence of definitively reported attributions of trauma or infection to TE formation, all patients were included in the analysis.

**Table 1:** General characteristics of the studies included in the systematic review

Author	Year	Type of article	Country	Number of included patients	Women (%*N*)	Age of epilepsy onset (years) mean (SD)	BMI mean (kg/m^2^) (SD)
Urbach et al.	2018	Case series	Germany	22	9 (40.9%)	23.4 (11.5)	27.5 (4.9)
Paule et al.	2019	Case series	Germany	15	5 (33.3%)	40.6 (20.5)	29.2 (8.5)
Tse et al.	2020	Case series	Australia	13	8 (61.5%)	24.9 (11.9)	27.1 (8.4)
Sandhu et al.	2022	Case series	USA	10	6 (60%)	32.0 (14.5)	31.4 (7.7)
Thottiyil et al.	2022	Case report	India	1	1 (100%)	21	23
Jagtap et al*	2022	Case series	India	9	2 (22.2%)	13.8 (3.8)	22.7 (4.4)
Tsalouchidou et al.	2023	Case series	Germany	24	10 (41.7%)	18.8 (10.8)	26.3 (5.9)
Buraniqi et al.	2023	Case series	USA	11	4 (36.4%)	10.1 (4.7)	23.9 (6.7)
Samudra et al.	2023	Case series	USA	22	18 (81.8%)	31.5 (11.0)	32.8 (8.1)

SD: Standard Deviation; BMI: Body Mass Index, *the information on sex was not reported separately for each patient (*n*=9) but only on average

### BMI and sex in relation to epilepsy onset

To examine the impact of BMI and sex on epilepsy onset, separate univariate logistic regression analyses were performed. Both models, one for BMI (*p* < 0.001) and another for sex (*p* = 0.023), demonstrated statistical significance, distinguishing patients experiencing epilepsy onset before and after the 25th year. BMI was a significant positive predictor (*p* < 0.001), indicating 15.3% higher odds of epilepsy onset with each unit increase. Regarding sex, female patients (*p* = 0.024) were associated with a 2.36-fold increase in odds of epilepsy onset after the 25th year. Subsequently, a bivariate logistic regression incorporating both BMI and sex showed an overall significant model (*p* < 0.001), explaining 23.4% of the variance (Nagelkerke´s R^2^ = 0.234). The overall classification accuracy was 69.5% with a sensitivity of 53.8% and a specificity of 81.8%. Notably, only BMI contributed significantly to the prediction of seizure onset (*p* < 0.0001), while sex did not (*p* < 0.181). Patients with increased BMI had a 1.133 times higher likelihood of presenting epilepsy onset after the 25th year of age (OR = 1.133, 95%-CI [1.060, 1.211]). Detailed coefficients and odds are available in Table 2.

**Fig. 2:**
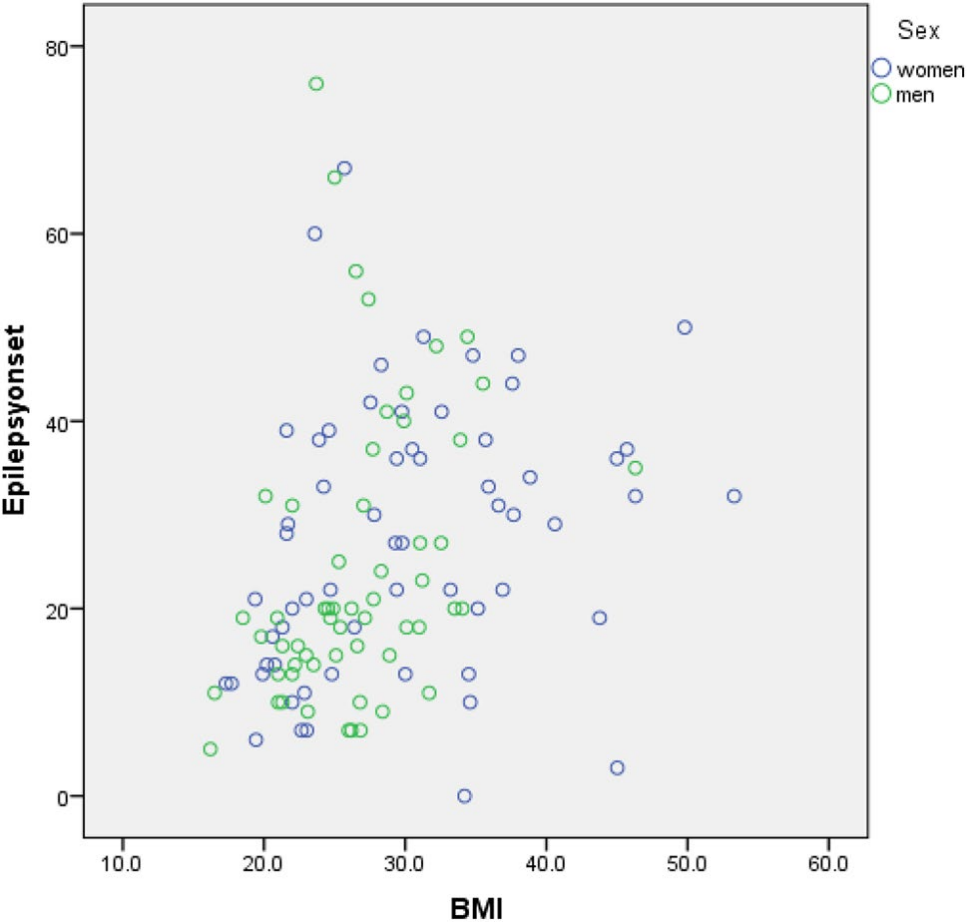
Figure showing the relationship between epilepsy onset in years and body mass index (BMI) in kg/m^2^ among female and male patients

A correlation analysis was conducted to explore the relationship between BMI and the age of epilepsy onset in the included patients. Spearman’s rho correlation coefficient revealed a significant positive correlation between BMI and epilepsy onset (rho = 0.448, *p* < 0.001). This finding indicates that higher BMI values are associated with a tendency for later onset of epilepsy in the studied population. The BMI distribution of patients in relation to the epilepsy onset is presented in Fig. 2.

Moreover, the relationship between BMI in male and female patients was examined. The mean BMI was 30.1 kg/m^2^ (SD = 8.7 kg/m^2^) and 26.5 kg/m^2^ (SD = 5.3 kg/m^2^) in the female and male patients, respectively. The BMI of the female patients was significantly higher than the BMI of the male patients (*p* = 0.008). Regarding the relationship between epilepsy onset and sex, the mean epilepsy onset was 27.3 years (SD = 14.4 years) and 24.2 years (SD = 15.4 years) in the female and the male patients, respectively, and did not differ significantly between the two groups (*p* = 0.26). The distribution of epilepsy onset in relation to the BMI among the female and male patients is presented in Fig. 2.

Finally, we examined the relationship between BMI and epilepsy surgery outcome. Of the included patients, 70.1% (*n* = 89) underwent epilepsy surgery. Among the surgically treated patients, epilepsy surgery outcome was reported separately in 75% (*n* = 67). The mean BMI among patients with favourable outcomes (Engel Class I and II) was 27.3 kg/m^2^ (SD = 7.7 kg/m^2^), while patients with less favourable outcomes (Engel Class III and IV) had an average BMI of 24.8 kg/m^2^ (SD = 2.2 kg/m^2^). There was no significant difference in BMI observed between patients with these outcomes (*p* = 0.076). Nevertheless, the average BMI among patients with favourable outcomes was higher than that of patients with less favourable outcomes, suggesting a potential trend and possible association between BMI and surgical outcomes.

### Signs of intracranial hypertension and bilateral TEs

Among the included patients, signs of ΙΙΗ were reported in 18.9% (*n* = 24), while 55.1% (*n* = 70) showed no indications of ΙΙΗ. Information on ΙΙΗ including secondary imaging signs was unavailable for 26% (*n* = 33) of the included patients. Additionally, the age of epilepsy onset differed significantly between patients with signs of ΙΙΗ (35.8 years, SD = 16.5) and those without reported signs (25 years, SD = 13.6), indicating a later onset in individuals with ΙΙΗ (*p* = 0.002).

In 30 out of 127 patients (23.6%), bilateral TEs were reported. There was a significant difference in the age of epilepsy onset between patients with bilateral TEs (mean age 32.1 years, SD = 18.7) and those with unilateral TEs (mean age 22.7 years, SD = 12.6), indicating a later epilepsy onset for individuals with bilateral TEs (*p* = 0.002). Furthermore, patients with signs of intracranial hypertension demonstrated a notably higher incidence of bilateral TEs (*p* < 0.001), suggesting a significant association between intracranial hypertension indicators and the presence of bilateral TEs.

**Table 2:** Model coefficients and odds of the binominal logistic regression analyses

Variable	Analysis	B	SE	Wald	*p*	OR	95% CI OR	
							Lower bound	Upper bound
BMI	Univariate	0.142	0.033	18.273	< 0.0001	1.153	1.080	1.230
Constant	Univariate	-4.360	0.951	21.036		0.013		
Sex	Univariate	0.857	0.381	5.072	0.024	2.357	1.118	4.971
Constant	Univariate	-1.551	0.618	6.297		0.212		
BMI	Bivariate	0.125	0.034	13.611	< 0.0001	1.133	1.060	1.211
Sex	Bivariate	0.551	0.412	1.789	0.181	1.735	0.774	3.893
Constant	Bivariate	-4.614	1.092	17.860		0.010		

*Notes* BMI, Body Mass Index, standard error; OR, odds ratio; CI, confidence interval; Significant at α = 0.05

## Discussion

While TEs are often considered potential epileptogenic lesions, the precise pathogenesis of TE-related epilepsy remains unclear. TEs are typically categorized as congenital, present from birth, or secondary, due to trauma or surgical conditions. The most commonly suggested pathomechanisms are the mechanical irritation of brain parenchyma or their coexistence with other epileptogenic lesions such as cortical dysplasia and neuronal heterotopia [9, 18, 19]. Although increased BMI is considered a risk factor for developing temporal encephaloceles in both epilepsy and non-epileptic patients, many articles on TE-related epilepsy present normal BMI in the majority of the reported patients [5, 8–10]. The development of TEs in patients with increased BMI has been attributed to a combination of factors, including a predisposing vulnerability of the skull base, which is further accentuated by the impact of the altered CSF dynamics and IIH of these patients, contributing secondary to the formation and development of these lesions.

In the present analysis, we found a significant correlation between elevated BMI and a later age of epilepsy onset among the included population with TE-related epilepsy. This suggests that, as the BMI increases, epilepsy occurs later in years. This correlation does not necessarily imply causation; therefore, this finding should not be interpreted as evidence of BMI’s protective mechanism leading to delayed epilepsy onset in patients with TE-related epilepsy. Instead, it supports the hypothesis of distinct underlying pathomechanisms of epileptogenicity [10] in individuals with increased BMI, influenced by BMI’s impact on the formation of TEs. Similarly, patients with signs of IIH exhibited a significantly more delayed epilepsy onset compared to those without, suggesting a potential association between ΙΙΗ and the progression of epilepsy onset over time. The gradual formation and development of TEs in patients with elevated BMI and IIH may be associated with milder and more gradual pathomechanisms of epileptogenesis compared to patients with normal BMI and congenital TEs.

While many case series present a higher prevalence in female patients [10] and others show opposite results [17], the current population of this analysis did not show any predominance in female patients. Conversely, the BMI was significantly higher in female than in male patients, while the mean age at epilepsy onset did not differ significantly between male and female patients. Moreover, a bivariate logistic regression was performed to examine the impact of BMI and sex in developing epilepsy after the 25th year of birth. This analysis showed that only increased BMI could predict a later epilepsy onset, while the sex of the population did not have a significant influence.

Furthermore, patients with signs of IIH showed an increased prevalence of bilateral TEs. Consistent with the findings of Paule et al. [8], individuals with bilateral TEs in this cohort had a delayed onset of epilepsy. These findings show a potential association between bilateral TEs and IIH, suggesting that bilateral TEs might even be an indicator for IIH.

Finally, the association between BMI and outcomes of epilepsy surgery has been examined by categorizing the outcomes as favourable (Engel I and II) and less favourable (Engel III and IV). The average BMI among patients with favourable outcomes was higher than that of patients with less favourable outcomes. Although the difference was not statistically significant, this finding suggests a potential trend between BMI and surgical outcomes worthy of further investigation.

The current analysis has some limitations. The role of BMI in the development of TE-related epilepsy has been discussed in recent years. Consequently, many earlier studies lack information about this characteristic within their published series, resulting in limited available reported cases. Therefore, due to the small sample size due to the limited reported cases in the literature, the results should be interpreted with caution. Moreover, in most of the included studies information about other potential TE-aetiologies, such as trauma or infection was not available.

## Conclusion

In conclusion, the present analysis shows an association between higher BMI and later epilepsy onset in TE-related epilepsy, implying potential distinct pathomechanisms in the epilepsy syndrome of patients with increased BMI. These results suggest the need for additional studies focused on exploring the underlying pathogenesis of TE-related epilepsy in individuals with obesity and TEs.

## Data Availability

The data of the review and analysis will be shared on request to any qualified investigator.
